# Building public engagement and access to palliative care and advance care planning: a qualitative study

**DOI:** 10.1186/s12904-024-01420-8

**Published:** 2024-04-12

**Authors:** Rachel Black, Felicity Hasson, Paul Slater, Esther Beck, Sonja McIlfatrick

**Affiliations:** 1 Institute of Nursing and Health Research, Ulster University, Belfast, BT15 1AD Northern Ireland; 2 Institute of Nursing and Health Research, Ulster University, Belfast, BT15 1AD Northern Ireland; 3 Institute of Nursing and Health Research, Ulster University, Belfast, BT15 1ED Northern Ireland; 4 Institute of Nursing and Health Research, Ulster University, Belfast, BT15 1ED Northern Ireland; 5 Institute of Nursing and Health Research, Ulster University, Belfast, BT15 1AD Northern Ireland

**Keywords:** Palliative care, Advance care planning, ACP, Social Ecological Model, Death literacy, Public engagement, Public health, Death and dying.

## Abstract

**Background:**

Research evidence suggests that a lack of engagement with palliative care and advance care planning could be attributed to a lack of knowledge, presence of misconceptions and stigma within the general public. However, the importance of how death, dying and bereavement are viewed and experienced has been highlighted as an important aspect in enabling public health approaches to palliative care. Therefore, research which explores the public views on strategies to facilitate engagement with palliative care and advance care planning is required.

**Methods:**

Exploratory, qualitative design, utilising purposive random sampling from a database of participants involved in a larger mixed methods study. Online semi-structured interviews were conducted (*n* = 28) and analysed using reflexive thematic analysis. Thematic findings were mapped to the social-ecological model framework to provide a holistic understanding of public behaviours in relation to palliative care and advance care planning engagement.

**Results:**

Three themes were generated from the data: “Visibility and relatability”; “Embedding opportunities for engagement into everyday life”; “Societal and cultural barriers to open discussion”. Evidence of interaction across all five social ecological model levels was identified across the themes, suggesting a multi-level public health approach incorporating individual, social, structural and cultural aspects is required for effective public engagement.

**Conclusions:**

Public views around potential strategies for effective engagement in palliative care and advance care planning services were found to be multifaceted. Participants suggested an increase in visibility within the public domain to be a significant area of consideration. Additionally, enhancing opportunities for the public to engage in palliative care and advance care planning within everyday life, such as education within schools, is suggested to improve death literacy and reduce stigma. For effective communication, socio-cultural aspects need to be explored when developing strategies for engagement with all members of society.

**Supplementary Information:**

The online version contains supplementary material available at 10.1186/s12904-024-01420-8.

## Background

It is estimated that globally only 14% of patients who require palliative support receive it [1]. The World Health Organisation (WHO) advocates for palliative care (PC) to be considered a public health issue and suggests earlier integration of PC services within the wider healthcare system is required [2]. However, research has shown that a lack of public knowledge and misconceptions about PC may deter people from accessing integrative PC services early in a disease trajectory [3]. Integral to good PC is the facilitation of choice and decision-making, which can be facilitated via advance care planning (ACP). Evidence suggests that ACP can positively impact the quality of end of life care and increase the uptake of palliative care services [4]. While ACP is commonly associated with end of life (EOL) care, it provides the opportunity for adults of any age to consider their wishes for future care and other financial and personal planning. However, there is evidence of a lack of active engagement in advance care planning (ACP) [5]. Recent research exploring knowledge and public attitudes towards ACP found just 28.5% of participants had heard the term and only 7% had engaged in ACP [6]. Barriers to engagement in ACP discussions have been found to include topics such as death and dying are considered a social taboo, posing an increased risk of distress for loved ones; and [6] a misconception that ACP is only for those at the end of life rather than future planning [7]. Therefore, there is a need for a public health approach to ACP, to enable and support individuals to engage in conversations about their wishes and make decisions surrounding their future care.

The need for a public health approach to PC, to tackle the challenges of equity and access for diverse populations, was noted in a recent Lancet paper [8]. This is further supported in a recent review, exploring inequalities in hospice care in the UK, Australia, New Zealand, and Canada which reported that disadvantaged groups such as those with non-cancer illnesses, people living in rural locations and homeless individuals had unequal access to palliative care [9]. They postulated that differing levels of public awareness in what hospice care provides, and to whom, was an influencing factor with variations in health literacy and knowledge of health services being present in both minority and socioeconomic groups [9].

Changes in how we experience death and dying have resulted in a shift away from family and community settings into healthcare settings. The Lancet commission exploring the ‘Value of Death’, suggests it has created an imbalance where the value of death is no longer recognised [10]. The commission’s report posits the need to rebalance death, dying and grieving, where changes across all death systems are required. This needs to consider how the social, cultural, economic, religious, and political factors that determine how death, dying, and bereavement are understood, experienced, and managed [10].

New public health approaches that aim to strengthen community action and improve death literacy, through increased community responsibility are reflected in initiatives, such as ‘Compassionate Communities’ and ‘Last Aid’ [11, 12]. However, a suggested challenge is the management of potential tensions that are present when attempting to conceptualise death in a way that mobilises a whole community [13]. Whilst palliative care education (PCE) can be effective in improving knowledge and reducing misconceptions, many PCE intervention studies, have focused on carers and healthcare professionals [14]. Initiatives such as ‘Last Aid’ attempt to bridge this gap by focusing on delivering PCE to the public, however, they are not embedded into the wider social networks of communities. It can be argued that public health campaigns, such as these are falling short by neglecting to use the full range of mass media to suit different ages, cultures, genders and religious beliefs [15]. Consequently, to understand what is required to engage the public successfully, the voice of the public must lead this conversation. Therefore, this study sought to explore public views on strategies and approaches to enable engagement with palliative care and advance care planning to help share future debate and decision making.

Within the last decades the delivery of PC and ACP have been increasingly medicalised and viewed as a specialist territory, however in reality, the care of those with life-limiting conditions occurs not only within clinical settings but within a social structure that affects the family and an entire community [16]. Therefore, death, dying and bereavement involve a combination of social, physical, psychological and spiritual events, therefore, to frame PC and ACP within a public health approach the response requires a shift from the individual to understanding the systems and culture within which we live. The Social Ecological Model (SEM) recognises the complex interplay between individual behaviours, and organisational, community, and societal factors that shape our acceptance and engagement. SEM provides a framework to understand the influences affecting engagement with PC and ACP and has been utilised as a lens through which the data in this study is explored.

## Methods

### Design

Qualitative research, using semi-structured interviews were adopted as this enabled an in-depth understanding of public views on strategies to enable engagement with PC. This research was part of a larger mixed-methods study [17]. Comprehensive Consolidated Criteria for Reporting Qualitative research (COREQ) were used [18](See Supplementary file 1).

### Sampling

A purposive random sampling method, using a random number generator, was adopted to recruit participants who consented to be contacted during data collection of a larger mixed methods study. Selected individuals were contacted by telephone and email to invite them to participate. Inclusion and exclusion criteria are outlined in Table 1. Interested individuals were provided with a participant information sheet detailing the aims of the study and asked to complete a consent form and demographic questionnaire.

**Table 1:** Inclusion/exclusion criteria

Inclusion criteria	Exclusion Criteria
Completed the 2022 Northern Ireland Life and Times Survey and provided contact details for further research	Not suffered bereavement within the last 6 months
Willing to participate and able to provide informed consent	
Aged over 18 years of age	
Able to speak and read English	
Access to telephone or Zoom/ Skype/MS Teams platforms	

A total of 159 participants were contacted, 105 did not respond, 21 declined and three were ineligible to participate. A total of thirty participants consented, however, two subsequently opted to withdraw prior to the interview.

### Data collection

Data was collected from December 2022 to March 2023 by RB. The qualitative interview schedule comprised four broad topic areas: (1) participants’ knowledge of PC and ACP; (2) sources of information on PC and ACP and current awareness of local initiatives for public awareness; (3) knowledge of accessibility to PC and ACP and (4) future strategies for promoting public awareness of PC and ACP, with a consideration of supporting and inhibiting factors. The interview schedule was adapted from a previous study on palliative care to incorporate the topic of ACP [3] (See Supplementary file 2). This paper reports on future strategies.

Participants were asked to complete a short demographic questionnaire prior to the interview to enable the research team to describe the characteristics of those who participated. These questions included variables such as age, gender, religion, marital status, behaviour relating to ACP and experience of PC.

Data was collected via online interviews conducted using the videoconferencing platform Microsoft Teams. Interviews lasted between 20 and 60 min and were recorded with participant consent. Data were stored on a secure server and managed through NVivo 12 Software.

### Data analysis

Qualitative data were transcribed verbatim automatically by Microsoft Teams and the transcripts were reviewed and mistakes corrected by the interviewer. All identifying information was removed. Transcripts were analysed using reflexive thematic analysis which involved a six-step process: familiarisation, coding, generating initial themes, developing and reviewing themes, refining, defining and naming themes, and writing up [19]. Themes were derived by exploring patterns, similarities and differences within and across the data in relation to participant’s views on the promotion of PC and ACP and the best ways to engage the public in open discussions.

The study explored the data through a SEM lens to provide a holistic framework for understanding the influences surrounding health behaviour change in relation to palliative care and advance care planning by mapping the findings to each of the SEM constructs.

The SEM for public health was conceptualised by McLeroy et al. [20]., and was based on previous work by Bronfenbrenner’s ecological systems theory [21]. The SEM looks to identify social-level determinants of health behaviours [22]. Five factor levels have been identified within the SEM; (1) Intrapersonal factors (2) Interpersonal processes (3) Institutional factors (4) Community factors and (5) Public policy [20]. In short, the SEM suggests that the social factors that influence health behaviours on an individual level are nestled within a wider complex system of higher levels. Current research literature has explored SEM as a model for understanding barriers and facilitators to the delivery of PC, adults’ preferences for EOL care and older adults’ knowledge and attitudes of ACP within differing socioeconomic backgrounds [23–25]. It has demonstrated the importance of a multilevel approach within these populations. However, there is a scarcity of research exploring strategies for public engagement with PC and/or ACP which are underpinned by SEM theory.

### Rigour

To ensure rigour in the analysis four members of the research team (RB, SM, FH, EB) independently reviewed the transcripts and were involved in the analysis and development of themes as a method of confirmability [26].

### Ethics

Ethical approval was gained from the University Research Ethics Filter Committee prior to commencing data collection. Participants provided written informed consent prior to the commencement of the interviews. They were advised of their right to withdraw, and the confidentiality and anonymity of all data were confirmed. All data was kept in accordance with the Data Protection Act (2018) [27].

## Results

All participants were white; 70% were female (n-19) and 70% were either married or cohabiting (n-19). The largest proportion of the sample 44% was aged under 50 years (n-12), with 22% aged between 50 and 59 (n-6) and 33% (n-9) aged between 60 and 84. Over half of the sample was employed (n-15), 15% were self-employed [4] whilst 26% were retired (n-7). Demographic data were missing for one of the included participants (see Table 2).

**Table 2:** Participant demographics

ID	Gender	Age	Employment status	Marital status	Ethnicity	Religious affiliation
**23609**	Male	30–39	Employed for wages	Cohabiting	White	Catholic
**31154**	Female	30–39	Employed for wages	Single never married	White	Protestant
**21647**	Female	18–29	Employed for wages	Single never married	White	Catholic
**21263**	Male	50–59	Self-employed	Single never married	White	No religion
**17852**	Male	40–59	Employed for wages	Married	White	Other Christian Denomination
**14876**	Female	50–59	Employed for wages	Separated	White	No religion
**32903**	Male	30–39	Employed for wages	Married	White	Protestant
**32288**	Female	60–69	Self-employed	Single never married	White	Agnostic
**37538**	Female	40–49	Employed for wages	Single never married	White	Catholic
**25103**	Female	50–59	Employed for wages	Married	White	Other religion or belief system
**24695**	Male	40–49	Employed for wages	Married	White	Protestant
**35036**	Male	80+	Retired	Married	White	Catholic
**28958**	Female	30–39	Employed for wages	Married	White	Catholic
**28907**	Female	40–49	Self-employed	Married	White	Other Christian Denomination
**12548**	Male	60–69	Retired	Married	White	Protestant
**29543**	Female	40–49	A student	Married	White	Catholic
**31832**	Female	30–39	Employed for wages	Cohabiting	White	Protestant
**23417**	Female	30–39	Employed for wages	Married	White	Protestant
**37172**	Male	60–69	Retired	Widower	White	Catholic
**34106**	Female	60–69	Retired	Married	Missing	No religion
**34022**	Female	60–69	Retired	Cohabiting	White	Protestant
**13697**	Female	50–59	Self-employed	Single never married	White	No religion
**25046**	Female	Missing	Employed for wages	Married	White	No religion
**19265**	Female	70–79	Retired	Married	White	No religion
**19874**	Female	Missing	Missing	Missing	Missing	Missing
**22964**	Female	50–59	Employed for wages	Married	White	Catholic
**13790**	Female	60–69	Employed for wages	Divorced	White	No religion
**25262**	Male	50–59	Retired	Married	White	Protestant

Responses to questions relating to ACP knowledge and behaviours found just 12 participants had heard of the term ACP prior to completing the Northern Ireland Life and Times Survey. Furthermore, none of the participants had been offered the opportunity to discuss ACP and none had prepared a plan of their wishes and preferences.

### Main findings

**Fig. 1:**
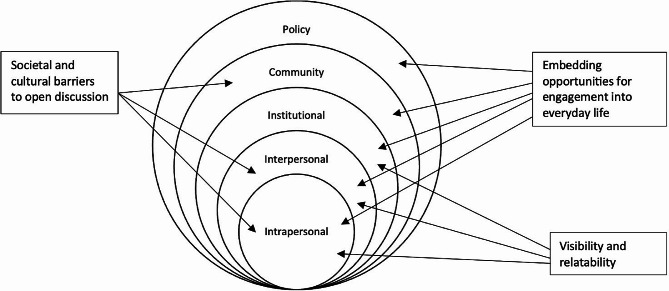
Thematic interaction within the Social Ecological Model levels

Three overarching themes were generated from the data: *‘Visibility and relatability’; ‘Embedding opportunities for engagement into everyday life’; ‘Societal and cultural barriers.* These findings were then mapped to the five social ecological model (SEM) levels (*individual; interpersonal; institutional; community; and policy*) to demonstrate the importance of a multilevel approach when seeking to engage the public around PC and ACP. See Fig. 1 for SEM construct mapping.

#### Theme 1: visibility and relatability

This theme relates to the suggestion that social taboo was a barrier to awareness and the mechanism to ameliorate this was visibility – in turn promoting reduction of stereotypes and promoting understanding and engagement. This posits the idea that the lack of understanding of PC is the root cause of much of the stigma surrounding it. The SEM construct mapping suggests a multilevel approach is required with intrapersonal (increased individual understanding), interpersonal (openness in discussion with friends and family through media normalisation) and institutional (health service policies for promotion and support) levels being identified.

Participants discussed how there is a lack of knowledge on what PC is, with many assuming that it was for people in the latter stages of life or facing end of life care. This highlighted the lack of individual education with participants suggesting that there should be more visibility and promotion on PC and ACP so that individuals are better informed.

*“So, it’s really um there needs to be more education, maybe, I think around it. So that people can view it maybe differently or you know talk about it a bit more. Yeah, probably demystifying what it is. This is this is what it is. This isn’t what it is. You know, this isn’t about um, ending your life for you, you know. And this is about giving you choices and ensuring that you know, you know people are here looking after you”(P37538F45)*.

However, there was a recognition that individual differences play a part in whether people engage in discussions. A number of participants explored the idea that some people just don’t want to talk about death and that for some it was not a subject that they want to approach. Despite this, there was a sense that increasing visibility was considered important as there will still be many people who are willing to increase their knowledge and understanding of PC and ACP.

*“I can talk about it, for example, with one of my sisters, but not with my mom and not with my other sister or my brothers. They just refuse point blank to talk about it…. some of them have done and the others have started crying and just shut me. Shut me off. And just. No, we don’t want to talk about that. So, it just depends on the personality, I suppose” (P14876F59)*.

The lack of knowledge and awareness of PC and ACP was suggested to be the attributed to the scarcity of information being made available at a more institutional level. For some participant’s, this was felt to be the responsibility of the health service to ensure the knowledge is out there and being promoted.

*“I think people are naive and they know they’re not at that stage and they don’t know what palliative care is, you know. It’s all like it’s ignorance. But our health service is not promoting this. Well in my eyes, they’re not promoting it whatsoever. And they should, they should, because it would help a hell of a lot of people*” (P37172M61).

*“I and I think it needs to be promoted by the point of contact, whether it’s a GP, National Health, whatever it might be, I think when they’re there, there needs to be a bit more encouragement to have that conversation” (P26495M43)*.

The lack of visibility within the general practice was discussed by several participants who said that leaflets and posters would be helpful in increasing visibility. One participant went as far as to say that a member of staff within a GP surgery would be beneficial.

*“I suppose the palliative care because it is a bit more personal. There should be even maybe a professional that you could talk to in your GP practice, or you know, like they have mental health practitioners now in GP practices. Maybe there are I don’t know if there is or not, but there should be maybe a palliative health practitioner that talks to people when they are at that stage of their life”* (P21647F29).

Participants also noted how there were generational differences in how people accessed health information. Many of the participants suggested that they would turn to the internet and ‘google’ for information, however, the suggestion was made that care should be taken to target awareness campaigns to different age groups via different methods to reduce disparities in technology skills such as those with less computer literacy.

“*I think a certain proportion of society need the visibility because they’re not always going to be self-sufficient enough to jump on the Internet even though you know we’re getting to the point now where the generational thing is. The generation have been brought up with the Internet and they’re obviously they go to it as the first point of call. But we still got the generation at the moment that don’t”. (P25046F-)*

One of the participants talked about ways to increase visibility via the use of the media, including social media, and the utilisation of famous faces.

*“Yeah, I think you know, they need to discuss it on Loose Women. You know, morning TV need to get on the bandwagon….But you know. It only takes like that one celebrity to mention it and then the whole media is jumping on the bandwagon.” (P19874F-)*.

The UK media coverage of other successful campaigns such as those highlighting mental health and bowel cancer were noted to have been particularly helpful in raising awareness.

*“if I think myself about the whole exposure that we have and as a society at the minute about mental health in general, you know a lot of the work on that has been done via social media. You know, celebrities hash tagging and talked about their experience. It’s OK to not be OK etcetera. And I feel like that is responsible for a lot of people who are now discussing their mental health” (P21647F29)*.

The sentiment expressed in the above quotes regarding increased visibility in the media also suggests that unless a topic seems relevant to an individual then they won’t engage with health promotion. This concept of relatability pertains to those aspects of human empathy where they can place themselves within a situation leading it to become more relevant to them.

Many participants discussed that using real-life stories on television and in campaigns would be an effective way to connect with the public and it would make PC and ACP more relatable and highlight the importance of thinking about it.

*“I think always what tends to be most effective is when it’s somebody that we could all relate to telling their own story (…)……I’m not too sure I know enough about what it involves, but really, like the consequences, that the consequences that people have suffered from not having done that. Not knowing what the wishes were, not having planned for it”. (P32288F62)*

Several participants discussed how the topics of PC and ACP were not something they would identify with as being relevant to them. The suggestion was that without it being an immediate concern, for example, if they were not approaching a certain age, then they would assume that they did not need to increase their knowledge about what PC or ACP involves.

*“You have to be able to relate to it in order to think, oh, yeah, that applies to me. You need to have something in which you identify with”. (P13697F51)*

#### Theme 2: embedding opportunities for engagement into everyday life

Throughout the discussions, there was evidence that participants felt that death literacy could be increased by providing more opportunities to gain knowledge about planning for future care and what PC involves. Education was highlighted as a potential pathway to engaging the public by targeting appropriate age groups and professions with relevant knowledge and skills. For some, this was thought to be best achieved through educating the youth and for others, the importance of educating those who are working in the healthcare profession was particularly salient. In addition, almost half of the participants suggested they would approach charity organisations for information, with participants advocating for education within secondary and tertiary levels and within community organisations. This data reflects an institutional-level construct within the SEM framework.

Educating younger generations on the topics of PC and ACP through open discussions in schools, and providing skills on how to have difficult conversations with loved ones were seen to be a valuable strategies.

“*young people don’t have that ability to accept and admit and bring it out into the open and I think they need to be perhaps encouraged more to do that through some kind of teaching in the school environment when they’re at a young and impressionable age*”. (P25046F-)

Due to the difficulties around having conversations about death, it was suggested that different healthcare professionals should be trained to have conversations with their patients.

*“Yeah, it’s like you think the discussions are difficult to broach for maybe health care professionals that you know, a difficult topic even for them to bring up. Well, if you’re working with someone who’s you know with a family and where things are quite distressed and very often it can be either, it can be the stress can cause a lot of friction and you know, decision making can be very difficult for people…., but just at every level, there’s, you know, possibility to be having conversations like that with people.”(P37538F45)*.

*“I suppose you could think about training some care professionals (…) there may be some way that as a second part of the person’s job or whatever that they’re trained so that people could go along and discuss ” (P19265F76)*.

Further to education for young people and healthcare professionals, there was the recognition that community organisations are perfectly positioned to educate the public in PC and ACP. One participant highlighted the missed opportunity to educate family members and carers through an existing programme on dementia. This is particularly pertinent to ACP due to the impact of cognitive decline on decision-making.

*“I went to zoom meetings for four weeks in a row with the Alzheimer’s Society. Umm regarding things to do with dementia. And you know, there was a week about your finances and things like that. I suppose. They never really talked about end of life care you know that sort of thing. Um I suppose it would have been useful had they you know, broached that subject as well. But it wasn’t, you know, there was more about looking after yourself, looking after the person. The symptoms of dementia and all this sort of thing, Alzheimer’s and then you know, um the financial and the help available to you. You know, but they didn’t mention about the end-of-life care and like the end result of dementia, I guess, is death. So, you know that that subject, you know, I was, I suppose to just those organizations that deal with um the issues of, like dementia or, as you say, all the rest for, you know, the rest of the diseases and that. You know to be up front and honest and say you know where this can lead and to make people you know, make people aware that there is a palliative care process that can be gone through.” (P19874F-)*.

Furthermore, the option of a helpline was suggested with reference made to other successful charity helplines such as the National Society for the Prevention of Cruelty to Children (NSPCC) and The Samaritans. Whilst it was acknowledged there were specialist support services available for people living with a terminal diagnosis, there was a sense that a generic information support helpline would be helpful.

*“You know, people need to be (aware)… they’re not alone. There is help out there. You know, you see your helpline, your children, NSPCC, your Samaritans. All on all these helplines, I have to think, I’ve never seen a helpline for palliative care or who you can contact. You don’t see things like that.” (P22964F52)*.

In addition to education, the suggestion that embedding ACP discussions into other more common aspects of future planning such as will making, and organ donation was postulated as a potential way to engage the public. This demonstrated clear links to the policy and institutional level constructs within SEM. Changes within organisational policies and public law to support individuals to consider future planning would promote better engagement on a wider societal level.

Participants suggested that they would like to see some of the conversations surrounding ACP introduced into workplace policies and guidelines, as well as through other legal discussions. They noted that conversations surrounding future planning already occur when discussing legal wills and workplace pensions. The potential to expand these discussions to include ACP with solicitors and in workplaces was seen to be a missed opportunity.

*“So maybe um around people who are making their wills and you know, you get will making services advertised and things like that. And I think once you get into your 30s and 40s, people start thinking about a will and things like that. So maybe aligned to something like that, you know would get younger people.” (P13790F67)*.

*“when people talk about the pension, you know so retirement, you know, to make people aware about this as well. You know, I would say that’s probably good ways to reach people” (P21263M54)*.

Current legislation and promotion regarding organ donation were discussed as being successful in engaging the public and therefore implied that the government should take a more active role in the promotion of ACP.

*“If you look at the way, sort of, government have been promoting like organ donation and that. You know that sort of thing. And then people have really bought into it and you know, and there’s a lot of positivity around it. So, I think that’s sort of similar approach would be good”. (P29453F40)*

*“You know, people talking about pension, pension plans and so on and it’s part of the natural life circle, you know. So, if it’s in connection with this, you know, so think about your future, make your plans …so probably in connection with organ donation and so on, you know, so I think they could be trigger points, you know,, people talking about this.” (P21263M54)*.

#### Theme 3: societal and cultural barriers to open discussion

In conversations surrounding why there was a lack of openness in discussion, participants postulated that a potential factor was the influence of cultural and societal norms. This was found to overlap in the SEM levels of intrapersonal, interpersonal and community.

Rural farming communities were highlighted as potentially being more isolated and traditional in their views around death and dying, whilst those with strong religious beliefs were seen to be less likely to engage in discussions.

*“You know, and there is a, I’m out in the countryside. Well, it’s (place name). So it’s a relatively rural sort of conservative place and it and it’s that… you know you’re tied to the land, you’re tied to the farm. This is your home and sending you away from it early it is seen as a bit of a shameful thing. So yeah, to try and educate folk and to try and speak into that I think would be really helpful because again, I’ve seen situations where probably the individual’s life, the end of life, has been made tougher because the family have fought to keep that person at home.…. We’re going to try and do it ourselves” (P26495M43)*.

*“I think it’s probably a lot to do with religion more than likely because people just like to hear, because we still are very much, you know, a lot is dictated by religion. So, people just want to leave stuff in God’s hands so they don’t want to have like…that would be the kind of where my mom and dad are coming from, you know, don’t interfere with it, blah blah blah. So, it’s a very gentle like kind of reminding them, you know, well, I think we do need to think about it. And I think people are afraid that they’ll make again that um it’s kind of euthanasia you know what I mean” (P37538F45)*.

Cultural differences between countries were also considered with some seeing other global communities approaching death as a celebration rather than something to shy away from.

*“I think in Northern Ireland we are, and the UK and probably the world in general. We are really poor at talking about, about death, and it has to be a positive thing to be able to talk more about death. You look at other cultures where you know. Death is treated differently, you know, even in Africa things where, you know, it’s a real celebration, whereas it’s seen so differently in in Northern Ireland” (P31154F35)*.

Furthermore, regions and countries that have experienced war and conflict were perceived by one participant as a potential barrier to engaging in subject matters which involve death.

*“I don’t I don’t know about other countries, but those that grew up maybe during the conflict here, maybe it’s just something that, you know it’s it’s a completely different mindset to them” (P23609M39)*.

Whilst it is unclear from the data why the participant felt the conflict might inhibit engagement with the subject of dying, it could be interpreted that the participant was suggesting that death is too morbid to engage with following a conflict period which saw numerous deaths. An alternative interpretation could be that people are desensitised to death and do not see death as something that an individual has autonomy over.

It was noted that in many modern societies communities are changing. People no longer interact with their neighbours in the way they used to which results in a reduced sense of community responsibility.

*“I don’t know that everyone is as neighbourly as they used to be. Northern Ireland I always perceived as being open door policy and everybody looked out for everyone else. But I think as a society has changed and it has changed in Northern Ireland, and it’s become becoming more closed (…) So I think we need to try and encourage that community um experience back again so that people are mindful of their neighbours and share that responsibility and making sure that everyone’s OK” (P25046F)*.

This sense of social isolation was discussed by one participant who referred to homeless people. They reflected on how current social structures may not be providing the information and support to this minority group and therefore should be considered when developing public health approaches to engaging them in PC and ACP education.

*“And if you look at homeless people on the streets, where do they get the information from? Where do they get the care, the information, the attention. I know there are Street workers that work, but I don’t know again to what extent in Northern Ireland compared with the likes of London” (P25046F)*.

### Mapping the findings to a social ecological model framework

Following thematic analysis each of the resulting themes were mapped to the five socioecological levels identified by McLeroy et al. (1988) for health promotion programmes. Construct mapping can be found in Fig. 1 below.

## Discussion

The findings from this study highlight the complexity of current public perceptions of palliative care and their views on effective engagement with PC and ACP. Within medicalised western culture there is a tendency to focus on the preservation of life, with conversations about death avoided. This has resulted in death becoming a taboo, raising fear and stigma where death is equated with failure. These social taboos that exist around death, dying and bereavement are posited to stem from the lack of awareness and understanding of PC and ACP and the resulting stigma of approaching these discussions. There was evidence of influencing factors on all SEM levels, which demonstrates the need for a multifaceted public health approach that uses not only behaviour change communication but also social change communication, social mobilisation and advocacy. It can be argued this reflects the key aspects outlined in Lancet Commission report on ‘Valuing Death’, which advocated for a ‘systems approach’ [10]. This systems approach is aligned to differing levels within the SEM and the different approaches the public have identified when seeking to build public engagement and access to palliative care. Three key aspects were noted: visibility, embedding opportunities for engagement in everyday life and societal and cultural influences.

It was clear from the analysis that a major factor associated with poor public engagement was the lack of visibility within the public domain, which was hindering both the normalisation of death and understanding that PC was more than just end of life care. The findings demonstrated different ways to address the lack of visibility, such as the use of targeted social media and wider publicity campaigns. Research to date has demonstrated that palliative care education is a useful tool in improving knowledge of, confidence in and attitudes towards palliative care amongst healthcare professionals and carers [14]. Similar results have been noted for the public when exploring the potential to promote palliative care through various media challenges such as YouTube and social media [28]. This does, however, raise questions around the quality and accuracy of information offered via the media, taking cognisance of whether some of the messaging may inadvertently be adding to misunderstanding, and thus a lack of public engagement.

Secondly, the findings indicated that experience at the individual level within a social context was noted as an important element when seeking ways to increase public engagement with PC and ACP. The experience of illness, dying and loss is often overlooked, therefore, this points to the potential value of community-based education approaches, with peers enabling experience-based exchange. Such interventions have been noted in the literature on the role of volunteers and education [29]. This reflects the need for an overall public health palliative care approach that seeks to empower individuals, families and communities to draw on their own resources and community supports to adapt and cope with death and dying [6, 30].

Thirdly, the findings from this study indicated the need for enhancing opportunities for engagement in PC and ACP within everyday life. Research indicates there is an appetite for people to talk about death, for example, in the UK, a recent YouGov ‘daily question’ survey reported 67% of adults who responded think the subject of death and dying should be talked about in schools [31]. This speaks to the need to consider schools, workplaces and key trigger points in life as times to consider engagement with PC and ACP. This reflects the overall need for death literacy in society to improve experiences at the end of life [10].

Finally, the importance of socio-cultural aspects for the public cannot be underestimated. Therefore, effective communication strategies need to be tailored to individuals, and communities and be culturally appropriate. This has been noted as an important aspect for specific communities, such as the Chinese diaspora, for example, but nuances around this for specific ethnic, political, religious, and geographical aspects need further consideration [32]. Cultural competence, defined as an understanding of how culture affects an individual’s beliefs, values and behaviour, is an important consideration [33]. A meta-analysis of 19 review articles, concluded that interventions to increase cultural competence in healthcare were effective in enhancing the knowledge, skills and attitudes of healthcare providers, leading to clinical benefits for patients/clients through improved access and utilization of healthcare [34]. The translation of such reviews for public engagement in PC and ACP warrants further exploration. It has been advocated that elements of cultural systems should be analysed with a socio-ecological framework [35]. Such consideration and integration of salient contextual cultural factors could assist public messaging and cultural communication, which would enhance more effective and sustainable public engagement in PC and ACP.

### Limitations

When considering potential limitations, it is pertinent to note that due to the sensitive nature of the topic the exclusion criteria restricted the sample to those who had not experienced a recent bereavement. This may have limited the ability to gain a wider perspective, as the views of the recently bereaved may have provided further nuanced insights into how best to engage the public. Furthermore, the participant sample was limited to those involved in a larger mixed-methods study. This may have introduced bias in relation to true knowledge and attitudes due to the participants having completed the survey questionnaire prior to the interviews.

## Conclusions

In conclusion, this qualitative study has provided insights into how the public would like to be engaged in PC and ACP. The findings highlighted that to build public engagement and access to palliative care and advance care planning a multifaceted public health approach is required. Discussions of death and dying remain difficult for many members of society, therefore, an increase in death literacy across all systems to reduce misperceptions surrounding PC and APC is needed, by increasing visibility and providing opportunities for the public to engage with PC and ACP within everyday life. Finally, socio-cultural aspects need consideration when developing strategies to ensure effective communication and engagement with all members of the community.

### Electronic supplementary material

Below is the link to the electronic supplementary material.

Supplementary Material 1

Supplementary Material 2

## Data Availability

The datasets analysed are not publicly available but are available from the corresponding author upon reasonable request.

## Abbreviations

ACP: Advance care plan
PC: Palliative care
PCE: Palliative care education
SEM: Social ecological model
