# Supplementary material for: Building public engagement and access to palliative care and advance care planning: a qualitative study
Source: BMC Palliat Care. 2024 Apr 12;23:98. doi: 10.1186/s12904-024-01420-8 (PMC11010379; doi:10.1186/s12904-024-01420-8)
Supplement: Supplementary file 1 — Supplementary Material 1 [file 12904_2024_1420_MOESM1_ESM.docx]

| **No** | **Item** | **Comments** |  |  |  |
| --- | --- | --- | --- | --- | --- |
| **Domain 1: Research team and reflexivity** |  |  | Reported on page number or not applicable (N/A) |  |  |
| Personal Characteristics |  |  |  |  |  |
| 1. | Interviewer/facilitator | Research Associate (Rachel Black) conducted the interviews |  |  |  |
| 2. | Credentials | The research team has extensive experience in qualitative health research (PhD/Prof) | N/A |  |  |
| 3. | Occupation | RB was a Research Associate at the time of the study | N/A |  |  |
| 4. | Gender | Four female and one male researcher | N/A |  |  |
| 5. | Experience and training | The research team has extensive experience in conducting qualitative health research | N/A |  |  |
| Relationship with participants |  |  |  |  |  |
| 6. | Relationship established | The research Associate (RB) had no prior relationship with participants. | N/A |  |  |
| 7. | Participant knowledge of the interviewer | The researcher introduced herself to the participants and explained the purpose of the research | N/A |  |  |
| 8. | Interviewer characteristics | The interviewer (RB) was not a Nurse/Healthcare professional but has an MSc in Health Psychology with knowledge of public health promotion | N/A |  |  |
| **Domain 2: study design** |  |  |  |  |  |
| Theoretical framework |  |  |  |  |  |
| 9. | Methodological orientation and Theory | Reflexive Thematic Analysis was used and mapped to Social Ecological theory constructs | 8 |  |  |
| Participant selection |  |  |  |  |  |
| 10. | Sampling | Purposive sampling with random number selection from residents in Northern Ireland that had taken part in the Northern Ireland Life and Times Survey and who had consented to being contacted for phase two. | 6 |  |  |
| 11. | Method of approach | Participants were approached via email and/or telephone | 6 |  |  |
| 12. | Sample size | Twenty-eight | 7 |  |  |
| 13. | Non-participation | How many people refused to participate or dropped out? Reasons? | 7 |  |  |
| Setting |  |  |  |  |  |
| 14. | Setting of data collection | Data was collected online via teleconferencing software - Microsoft Teams. | 7 |  |  |
| 15. | Presence of non-participants | None | N/A |  |  |
| 16. | Description of sample | Residents of Northern Ireland, aged over 18 who took part in the 2022 Northern Ireland Life and Times Survey. | 6 |  |  |
| Data collection |  |  |  |  |  |
| 17. | Interview guide | An interview schedule was developed with 4 broad topic areas. All interviews covered these main questions but there was variation depending on the participant responses. | 7 |  |  |
| 18. | Repeat interviews | No | N/A |  |  |
| 19. | Audio/visual recording | All Interviews were videorecorded and later transcribed |  |  |  |
| 20. | Field notes | Brief field notes were made following the interviews | N/A |  |  |
| 21. | Duration | Interviews lasted between approximately 20-60 minutes |  |  |  |
| 22. | Data saturation | No | N/A |  |  |
| 23. | Transcripts returned | No | N/A |  |  |
| **Domain 3: analysis and findings**z |  |  |  |  |  |
| Data analysis |  |  |  |  |  |
| 24. | Number of data coders | RB coded the data and developed themes. Transcripts, coding and themes were shared with all members of the research team and discussed for credibility | 9 |  |  |
| 25. | Description of the coding tree | No | N/A |  |  |
| 26. | Derivation of themes | Themes were derived from the data | 8 |  |  |
| 27. | Software | Nvivo12 |  |  |  |
| 28. | Participant checking | No | N/A |  |  |
| Reporting |  |  |  |  |  |
| 29. | Quotations presented | Yes participant quotes were used to demonstrate findings. Quote sources are identified by a P plus digit = interviewee identity, M = Male; F= Female and age range to protect participant confidentiality | 12-24 |  |  |
| 30. | Data and findings consistent | There was consistency between data and findings | 12-24 |  |  |
| 31. | Clarity of major themes | Yes all main themes are clearly identified | 12-24 |  |  |
| 32. | Clarity of minor themes | N/A |  |  |  |
