# Supplementary material for: Building public engagement and access to palliative care and advance care planning: a qualitative study
Source: BMC Palliat Care. 2024 Apr 12;23:98. doi: 10.1186/s12904-024-01420-8 (PMC11010379; doi:10.1186/s12904-024-01420-8)
Supplement: Supplementary file 2 — Supplementary Material 2 [file 12904_2024_1420_MOESM2_ESM.docx]

**SUPPLEMENTARY INFORMATION 2**

**Interview Schedule**

**Study Title: BUILDING PUBLIC ENGAGEMENT AND ACCESS TO PALLIATIVE CARE AND ADVANCE CARE PLANNING: A QUALITATIVE STUDY**

**Topic themes & questions**

*General knowledge of palliative care*

- Could you please describe for me what you think palliative care is?
- Where do you think palliative care takes place?
- Views and opinions of palliative care.

*General knowledge of ACP*

- Could you please describe for me what you think ACP is?
- When do you think ACP takes place?
- Views and opinions of ACP.

# *Knowledge and information*

- If you needed information about palliative care/ACP, where would you look for it, or whom would you ask?
- Are you aware of any local initiatives in community centres or libraries which discuss ACP/palliative care?
- Have you attended any and can you tell me if it changed how you thought about ACP afterwards?

# *Accessibility*

- Where do you think people go to get ACP/ palliative care services?

*Future Strategies*

- What do you think are the supporting factors for promoting public awareness of palliative care/ACP?
- What do you think are the inhibiting factors for promoting public awareness of palliative care/ACP?
- What could be done to promote more openness in discussion (and to inform future strategies)?
- Would you like to see palliative care/ACP publicly promoted? Please explain. If yes how would this be achieved?
